# Elevated Level of PKMζ Underlies the Excessive Anxiety in an Autism Model

**DOI:** 10.3389/fnmol.2019.00291

**Published:** 2019-11-28

**Authors:** Xiaoli Gao, Rui Zheng, Xiaoyan Ma, Zhiting Gong, Dan Xia, Qiang Zhou

**Affiliations:** ^1^School of Chemical Biology and Biotechnology, Peking University Shenzhen Graduate School, Shenzhen, China; ^2^Department of Anatomy, College of Preclinical Medicine, Dali University, Dali, China; ^3^Department of Child Healthcare, Shenzhen Children’s Hospital, Shenzhen, China; ^4^State Key Laboratory of Chemical Oncogenomics, School of Chemical Biology and Biotechnology, Peking University Shenzhen Graduate School, Shenzhen, China

**Keywords:** autism, valproic acid, protein kinase Mζ, anxiety, basolateral amygdala

## Abstract

Anxiety affects the life quality of a significant percentage of autism patients. To understand the possible biological basis of this high anxiety level, we used a valproic acid (VPA) model of autism. Anxiety level is significantly higher in VPA-injected mice, at both P35 and P70. In addition, protein kinase Mζ (PKMζ) level in the basolateral amygdala (BLA) is significantly higher in VPA mice at both ages. Consistent with this finding, infusion of a PKMζ-blocking peptide z-pseudosubstrate inhibitory peptide (ZIP) into BLA significantly reduced anxiety levels in VPA mice. Furthermore, viral overexpression of PKMζ in the BLA led to elevated anxiety level in Wild Type (WT) mice, with concomitant higher intrinsic excitability of BLA excitatory neurons. Altogether, our results indicate a key contribution of BLA PKMζ level to anxiety, especially in autism; and this finding may provide a further understanding of the pathogenesis as well as treatment of anxiety symptoms in autism patients.

## Introduction

Anxiety is a prominent feature in patients with autistic spectrum disorders (ASDs) since it has been reported that 80% of ASD patients exhibit significant anxiety level (Sharma et al., 2014; Opoka and Lincoln, 2017). There is considerable evidence that children and adolescents with ASD are at increased risk of anxiety and anxiety disorders (Skokauskas and Gallagher, 2010). It has been estimated that nearly 40% of ASD children and adolescents may have elevated levels of anxiety or at least one type of anxiety disorders (van Steensel et al., 2011). Hence, better understanding of the biological underpinning of anxiety in ASD is of great importance for both pathogenesis and treatment.

Previous studies have shown that mice exposed to valproic acid (VPA) prenatally around embryonic day (E) 12.5 display abnormalities in their neurological and behavioral development recapitulates many of the core symptoms of ASD, including impaired social behaviors (Wu et al., 2017), increased repetitive behaviors (Wang et al., 2018) and increased excitatory to inhibitory ratio (Banerjee et al., 2014), elevated physiological and behavioral measures of anxiety (Edalatmanesh et al., 2013; Kerr et al., 2013; Olexová et al., 2016), and enhanced responsivity to sensory stimulation (Fontes-Dutra et al., 2018). Neural circuits in key brain regions implicated in ASD, such as the amygdala, are altered in VPA model animals and thus they are a useful model to investigate circuitry malfunctions in emotional and behavioral abnormalities, especially anxiety.

Abnormal functioning of the amygdala has long been implicated in the etiology of ASD (Kerr et al., 2013), and it is of the major brain structures contributing to anxiety (Bertelsen et al., 2017). Behavioral phenotypes associated with anxiety disorders are accompanied by alterations in GABAergic transmission in the amygdala (Wu et al., 2018), such as low GABA levels (Nemeroff, 2003; Muller et al., 2007). Thus, hyperexcitability of amygdala may directly contribute to the genesis of anxiety. Elevated expression of GluN2A- and GluN2B-NMDA receptors and augmented long-term potentiation (LTP) were found in neocortex of VPA mice indicating increased excitatory synaptic functions (Wang et al., 2018).

To further identify potential mediators of this hyperactivity in the amygdala, we decide to focused on protein kinase Mζ (PKMζ), which is an atypical protein kinase C (PKC) isoform implicated in the protein synthesis-dependent maintenance of LTP and memory storage in the brain (Chen et al., 2014; Xue et al., 2015). The constitutive activity of PKMζ has been shown to maintain LTP as inhibiting its activity diminishes LTP (Naik et al., 2000; He et al., 2011). PKMζ is mainly expressed in the pyramidal cells in the hippocampal region and neocortex, granular cells in dentate gyrus, and Purkinje cells in cerebellum (Naik et al., 2000). In hippocampus (HPC), PKMζ is distributed widely in the soma and dendrites, with no expression in the axons of the HPC, and expression is highest in the postsynaptic region and dendrites (Kwapis and Helmstetter, 2014). PKMζ is highly expressed in the endoplasmic reticulum, but very low in the Golgi and mitochondria (Howell et al., 2014). Null mice of *Prkcz* that lose both PKMζ and PKCζ activity exhibit normal behavior in shuttle box test, basal motor functions and sensory perception, but they display reduced anxiety-like behavior (Lee et al., 2013), suggesting a role of PKMζ in anxiety.

In this study, we found that the prominent anxiety level in VPA mice is associated with a high expression of PKMζ in the basolateral amygdala (BLA). Functional inhibition of PKMζ significantly reduces anxiety level in VPA mice while elevation of PKMζ level *via* viral overexpression in BLA in wild type (WT) mice results in enhanced anxiety and higher intrinsic neuronal excitability. Thus, PKMζ level in BLA is highly relevant to the anxiety level, and may contribute to the pathological anxiety in autism patients.

## Materials and Methods

### Animals

ICR WT mice were purchased from Guangdong Medical Laboratory Animal Center (Guangdong, China), and all experiments have been approved by the Peking University Shenzhen Graduate School Animal Care and Use Committee and were in accordance with the ARRIVE guidelines on the Care and Use of Experimental Animals. Male and female animals were fed separately and housed in groups of 4–5. All mice were maintained under standard laboratory conditions at 22 ± 2°C, with 50 ± 10% relative humidity and on a 12 h-light/dark cycle, with food and water made available *ad libitum*.

Female mice weighing 40–60 g and male mice weighing 40–70 g ICR mice were used. Before any experimental procedure was carried out, animals were acclimated for 1 week in the experimental rooms. Their fertility cycle was controlled, and they were allowed to mate overnight when females were in a pro-estrus state. Vaginal smears on glass slides were examined on the following morning; and if spermatozoa were found, it was designated as first day of pregnancy. Each pregnant mouse was then housed separately and divided into control and VPA-treated groups. VPA (Cat. No. p4543, Sigma-Aldrich, UK) was dissolved in 0.9% saline at a concentration of 250 mg/ml. Females received a single intraperitoneal injection of 600 mg/kg sodium valproic (VPA) on E12.5 day after conception, and control females were injected with the same amount of saline at the same time point. Females were housed individually and allowed to raise their own litters. The number of viable offspring born in both groups: control and VPA-treated was normal. All animals exposed to VPA during gestation developed a characteristic “kink” in their tails, which was easily distinguishable from the aged-matched controls. The offspring were weaned on postnatal day (PND) 21. All subsequent experiments were performed only on the male offspring.

### Behavioral Testing

#### Open Field

Open field test was conducted on Day 35 (P35) and adult (9–10 weeks, P70). Mice were allowed to acclimate for 1 h before testing in a quiet room under adjusted lighting. A square wooden box (100 cm × 100 cm × 40 cm) was used for this locomotor activity test. The floor area was divided into 25 blocks of equal size with nine blocks making up the center grid. Mice were allowed to acclimate in the box for 5 min and then placed inside the central block and its movements monitored with a video camera for 10 min. The number of blocks that mice passed through (cross grid) and the frequency of straight upward movements (vertical) were recorded. Open field was thoroughly cleaned with 70% alcohol between test animals.

#### Elevated Plus Maze (EPM) Test

The elevated plus maze (EPM) apparatus consisted of two open arms (30 × 5 cm), two closed arms of the same size with 15 cm high walls and a center platform (5 × 5 cm). The apparatus was elevated to a height of 35 cm above the test room floor. Mice were placed in the test room to habituate for 1–2 h. Mice were placed in the central area facing one of the open arms at the start of the test. Time in open arm and number of entries to open arm were recorded for 300 s using ANY-maze software. The apparatuses were cleaned with 75% alcohol after each test.

#### Shuttle (Light/Dark) Box

Shuttle box consisted of two compartments with different illumination intensity: a light chamber (295 lx) and a dark chamber (0 lx). They were of the same size and shape (21 × 21 × 25 cm) and separated by a Plexiglas wall (21 × 25 cm). A hole of 3 × 5 cm at the bottom of separating wall connected the two chambers. Mice were allowed to move freely between these two chambers. At the start of the test, mice were placed inside the dark chamber. Locomotion was recorded using a camera placed above the shuttle box, and time spent by each mouse in the light box was measured manually. Shuttle box was cleaned with 30% isopropanol after each test.

### Western Blot Measurements

Western blotting was used to examine the expression of PKMζ in BLA, HPC and medial prefrontal cortex (mPFC). Tissues were collected, homogenized in RIPA buffer containing 1 mM PMSF (Bi Yun Tian, China), 25 μM leupeptin (Sigma), and 1 μg/ml aprotinin (Sigma), centrifuged at 4°C for 0.5 h at 13,000 *g*, with the supernatant collected. Total protein lysates made from different encephalic regions were mixed with SDS gel-loading buffer and heated for 5 min at 100°C. Samples (15 μg protein in each group) were separated on 12% SDS-PAGE gels (Invitrogen, Carlsbad, CA, USA), and transferred to polyvinylidene difluoride membranes (Millipore, Bedford, MA, USA). The membranes were blocked for 1 h at room temperature with 5% nonfat milk in TBST (TBS containing 0.05% Tween 20) and then probed with specific primary antibody (anti-PKMζ, 1:2,000, Cat. No. JH6065, Covance, UK, Volk et al., 2013) for overnight at 4°C. A horseradish peroxidase (HRP)-conjugated secondary antibody was then added for 2 h at room temperature. Immuno-positive PKMζ bands were scanned and densitometrically analyzed by automated ImageJ software (NIH Image, Version 1.61), and their total protein densities were expressed relative to GAPDH signals.

### Peptide Infusion

To inhibit the activity of PKMζ in the BLA, mice were deeply anesthetized with isoflurane on the day of surgery. The stereotaxic coordinates for BLA were AP −1.4 mm and ML ±4.0 mm and DV −5.08 mm. The PKMζ inhibitor z-pseudosubstrate inhibitory peptide (ZIP) was dissolved in sterile saline and infused at a concentration of 10 mM. ZIP or saline were infused into BLA (400 nl per hemisphere) at a rate of 80 nl/min. The injection needle was left in place for an additional 5 min.

### Viral Transfection

To overexpress PKMζ in the BLA, mice were deeply anesthetized with isoflurane and injected with virus. Three-hundred nanoliter of rAAV-hSyn-GFP-pA virus (Brain VTA Technology Company Limited, Wuhan, China) at an injection speed of 80 nl/min was injected bilaterally in the BLA area (BLA; −1.4 AP, ±4.0 ML, −5.08 DV; from Bregma). Five-hundred nanoliter of rAAV-hSyn- PKMζ-pA virus (Brain VTA Technology Company Limited, Wuhan, China) was injected in the BLA unilaterally using a micro syringe pump. Behavioral experiments were conducted 4 weeks after this procedure.

### Immunohistochemistry

To examine whether there is sufficient overexpressed PKMζ in the parvalbumin (PV)-positive neurons, we examined the overlap between the expressed of AAV-hSyn-PKMζ-GFP and PV staining in brain sections. Mice brains were fixed with 4% (vol/vol) paraformaldehyde (PFA), dehydrated with gradient sucrose (20% and 30%) and embedded in optimal cutting temperature compound (OCT). The embedded tissues were then cut sagittal into 30 μm-thick sections using a freezing-sliding Microtome (Leica, Germany) as described previously (Zheng et al., 2017). Crysections were permeabilized in phosphate buffer containing 0.5% Triton X-100 (PBST), and incubated with primary antibodies (anti-PV, 1:500, ab11427, Abcam, UK) in blocking solution and incubated at 4°C overnight. Primary antibodies were detected using Alexa-Fluor fluorescent dye conjugated secondary antibodies (anti-rabbit; Alexa Fluor 546, A11035, Invitrogen, UK). Sections were then counterstained with DAPI for 10 min. For analysis and quantification of immunoreacted areas, sections were imaged using confocal microscopy (Olympus, Tokyo, Japan).

### Electrophysiological Recordings

Live brain slices were acutely prepared from mouse brains as previously described (Yao et al., 2018). Briefly, mice were anesthetized with sodium pentobarbital (1%), with brain quickly removed from the skull and transferred into ice-cold cutting artificial cerebrospinal fluid (ACSF) containing (in mM): 110 choline chloride, 25 D-glucose, 25 NaHCO_3_, 11.6 sodium ascorbate, 7 MgSO_4_, 3.1 sodium pyruvate, 2.5 KCl, 1.25 NaH_2_PO_4_, and 0.5 CaCl_2_. The coronal brain slices (400 μm) were cut using a DTK-1000 tissue slicer (DTK, Japan) in the cold cutting solution. The brain slices containing BLA recovered at 35.2°C for 30 min, with ACSF containing (in mM): 127 NaCl, 25 NaHCO_3_, 25 D-glucose, 2.5 KCl, 2 CaCl_2_, 1.25 NaH_2_PO_4_, and 1 MgCl_2_. Then, brain slices were incubated at room temperature for 1 h before recording. The slices were transferred into the recording chamber on an Olympus microscope (BX51WI) equipped with light and fluorescence illumination. The slices were immersed at room temperature with oxygenated ACSF (3 ml/min). Recording pipettes (3–5 MΩ) were filled with internal solution containing (in mM): 128 potassium gluconate, 10 NaCl, 10 HEPES, 4 Na_2_ATP, 2 MgCl_2_, 0.5 EGTA, 0.4 NaGTP. All solutions used for electrophysiology were equilibrated with 95% O_2_/5% CO_2_.

To measure excitability, recording in current-clamp mode was used. Neurons were injected with a 500 ms-long current pulse every 4 s. Data were acquired using HEKA EPC10 double patch clamp amplifier (HEKA). Signals were acquired at a sampling rate of 10 kHz and filtered at 2 kHz. Neurons with holding current larger than −200 pA (at −60 mV) were excluded from data analysis.

### Data Analysis

Data were analyzed using GraphPad Prism software. Statistical analysis was performed using unpaired *t*-test, paired *t*-test, One-way or Two-way Repeated Measures ANOVA (One-way or Two-way RM ANOVA) followed by Bonferroni post-test which were specifically stated in the “Results” section. All results were shown as Mean ± SEM. *P* < 0.05 was considered statistically significant.

## Results

### High Innate Anxiety in the VPA Mice

As mentioned in the “Introduction” section, increased anxiety has been reported in VPA models. Thus, we first tested whether anxiety is higher in our VPA mice and if so when this change occurs. Innate anxiety was measured in P35 and P70 VPA mice using light-dark shuttle box and EPM. The reason for not examining mice younger than P35 is that anxiety cannot be reliably measured at those ages under our experimental conditions.

**Figure 1:**
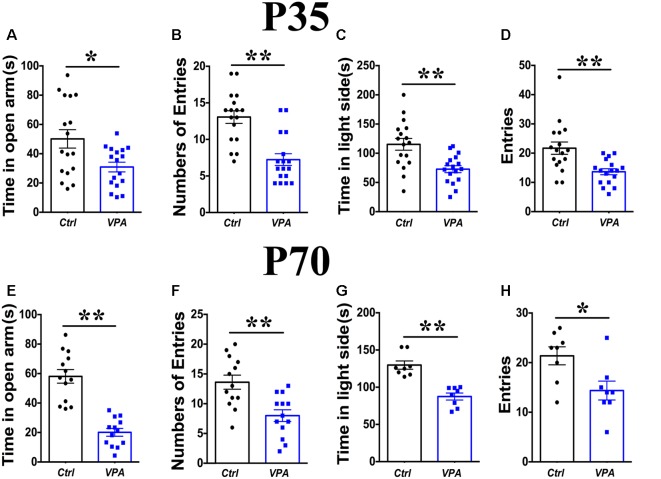
Elevated anxiety in valproic acid (VPA) mice. Time in the open arms **(A)** and numbers of entries to the open arms **(B)** on the elevated plus maze (EPM) was significantly reduced in P35 VPA mice compared to the control mice. Time in the light box **(C)** and entries to the light box **(D)** was significantly reduced in P35 VPA mice compared to the control mice. Time in the open arms **(E)** and numbers of entries to the open arms **(F)** on the EPM was significantly reduced in P70 VPA mice compared to the control mice. Time in the light box **(G)** and entries to the light box **(H)** was significantly reduced in P70 VPA mice compared to the control mice. *P < 0.05, **P < 0.01.

The EPM test showed elevated anxiety in VPA mice of both P35 and P70: (1) shorter time spent in the open arms (Figure 1A, P35, *P* < 0.05; Figure 1E; P70, *t*_(28)_ = 4.68, *P* < 0.01, *n* = 17 mice); and (2) less times entering into the open arms (Figure 1B, P35, *P* < 0.01; Figure 1F, P70, *P* < 0.05, *n* = 17). The above comparisons were made between VPA and vehicle-injected mice.

Measurements in the light-dark shuttle box also indicated elevated anxiety in VPA mice at P35 and P70: (1) less amount of time in the light box (Figure 1C, P35, *P* < 0.05; Figure 1G, P70, *P* < 0.01, *n* = 17); and (2) fewer times shuttling between light and dark boxes (Figure 1D, P35, *P* < 0.01; Figure 1H, P70, *P* < 0.05, *n* = 17). Taken together, these results indicate significant anxiety in the VPA mice of both adolescence and adult, consistent with prior studies and suggest that the above measurements can be used reliably to measure anxiety level in VPA model mice.

### Selective Elevated PKMζ Level in the BLA, HPC and mPFC of VPA Mice

**Figure 2:**
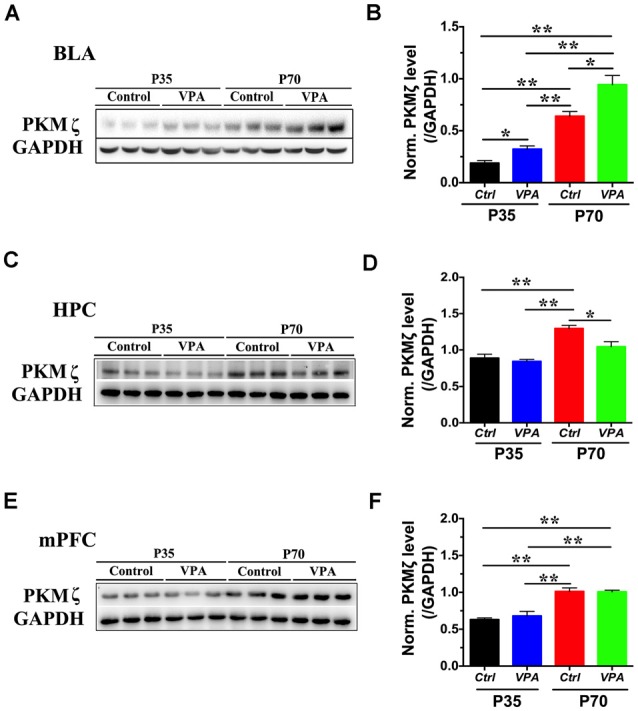
The level of protein kinase Mζ (PKMζ) in VPA mice. **(A)** Sample images of WB analysis of PKMζ level in the basolateral amygdala (BLA), at both P35 and P70. **(B)** Quantification showed significant elevation in PKMζ levels in both P35 and P70 VPA mice compared to control mice. **(C)** Sample images of PKMζ level in the hippocampus (HPC), at both P35 and P70. **(D)** Quantification showed significant reduction in PKMζ levels in P70 VPA mice compared to control mice. **(E)** Sample images of PKMζ level in the medial prefrontal cortex (mPFC) measured using WB, for both P35 and P70 mice. **(F)** Quantification showed no difference in PKMζ levels between VPA and control mice, at both P35 and P70. *n* = 3, *P < 0.05, **P < 0.01.

PKMζ, an isoform of PKC with persistent activity, is involved in anxiety associated with pain (Zhang et al., 2016; Du et al., 2017). In addition, reducing PKMζ activity has been found to be anxiolytic in a PTSD model (Ji et al., 2014). To examine whether PKMζ level is significantly altered in VPA mice, we used Western blot from VPA mice of P35 and P70, in three major brain regions that are involved in anxiety, the BLA, HPC and mPFC. A significant elevation in PKMζ level was seen in BLA of both P35 and P70 VPA mice, compared to vehicle-injected (Veh) mice (Figures 2A,B; *P* < 0.05, *P* < 0.01, *n* = 3). In VPA mice, the level of PKMζ was significantly increased in P70 mice compared to P35 mice (Figures 2A,B; *P* < 0.01, *n* = 3). In HPC, PKMζ level in P70 mice was significantly lower in the VPA mice compared to Veh group (Figures 2C,D; *P* < 0.05, *n* = 3), but there was no significant difference in PKMζ level between VPA mice and Veh mice at P35 (Figures 2C,D; *P* = 0.486, *n* = 3). In the mPFC, no significant difference in the PKMζ level was found between the VPA and Veh mice, at both P35 and P70 (Figures 2E,F; *P* = 0.4911, *P* = 0.9278, *n* = 3). Taken together, there is a significant and selective elevation in PKMζ level in the BLA of VPA mice at both P35 and P70 compared to Veh group.

### Reducing PKMζ Activity Reduces Anxiety in the VPA Mice

**Figure 3:**
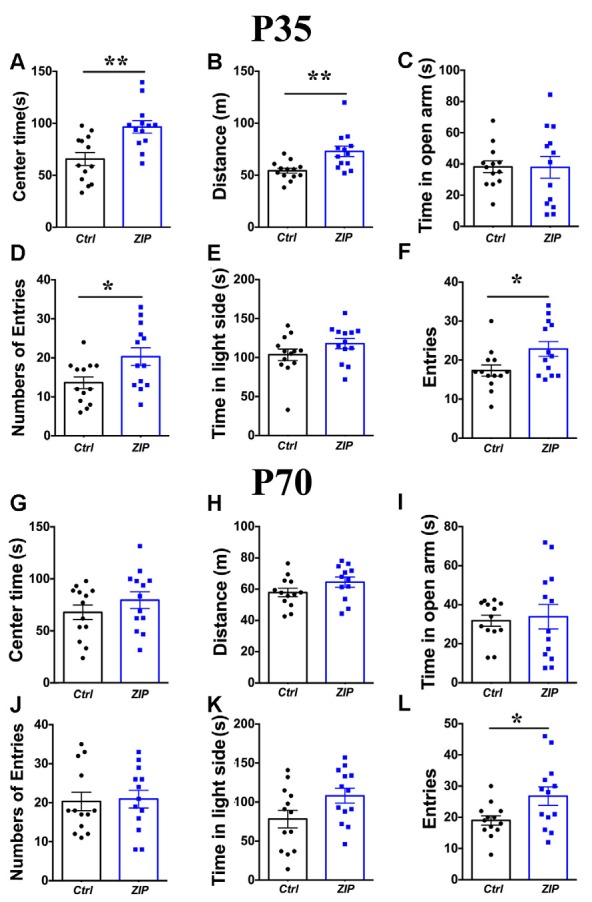
Reducing PKMζ activity with z-pseudosubstrate inhibitory peptide (ZIP) decreased anxiety in VPA mice. Time in the center **(A)** in the open field test were significantly increased after infusion of ZIP in the P35 VPA mice compared to those infused with Vehicle. Distance traveled **(B)** in the open field test were significantly increased after infusion of ZIP in the P35 VPA mice compared to those infused with Vehicle. Time in the open arm **(C)** was not altered while numbers of entries to the open arms **(D)** were significantly increased in the P35 VPA mice compared to those infused with vehicle. Time in the light box **(E)** was not affected while entries to the light box **(F)** was significantly increased in the P35 VPA mice compared to those infused with vehicle. Time in the center **(G)** in the open field test were not significantly altered after infusion of ZIP in the P70 VPA mice compared to those infused with vehicle. Distance **(H)** traveled in the open field test were not significantly altered after infusion of ZIP in the P70 VPA mice compared to those infused with vehicle. Time in the open arm **(I)** was not altered and numbers of entries to the open arms **(J)** were not significantly altered in the P70 VPA mice compared to those infused with Vehicle. Time in the light box **(K)** was not affected while entries to the light box **(L)** was significantly increased in the P70 VPA mice compared to those infused with vehicle. *P < 0.05, **P < 0.01.

One likely consequence of higher expression of PKMζ is higher activity of PKMζ. To further understand whether elevated PKMζ has a direct influence on the anxiety level in VPA mice, we inhibited its activity using a well-established method, a short peptide ZIP. Previous studies demonstrated the efficacy of ZIP in reducing the activity of PKMζ and erasure of formed memory (Lin et al., 2013). Bilateral infusion of ZIP into BLA of 30 min prior to testing showed significantly higher time in the center area (Figure 3A; *P* < 0.01, *n* = 13 mice), and there was also a significant increase in locomotion as revealed by longer distance traveled (Figure 3B; *P* < 0.01, *n* = 13). In the EPM test, there was no significant increase in the open arm time (Figure 3C; *P* = 0.97, *n* = 13), while numbers of entries to the open arms were significantly increased in the P35 VPA mice compared to those infused with vehicle (Figure 3D; *P* < 0.05, *n* = 13). In addition, the time in the light side was not altered (Figure 3E; *P* = 0.16, *n* = 13), and entries to the light side (Figure 3F; *P* < 0.05, *n* = 13) was significantly higher in the ZIP-infused VPA mice, compared to vehicle-injected mice.

In contrast, the same infusion did not result in significant changes in anxiety in P70 VPA mice. We found no significant impact of ZIP on the center time (Figure 3G; *P* = 0.28, *n* = 13), and distance traveled was not altered (Figure 3H; *P* = 0.12; *n* = 12). We also found that neither time in the open arms (Figure 3I; *P* = 0.77, *n* = 13) nor numbers of entries to the open arms (Figure 3J; *P* = 0.85, *n* = 13) in the EPM was altered; nor was time in the light side of shuttle box (Figure 3K; *P* = 0.06, *n* = 13). The only significant change was entries to the light box (Figure 3L; *P* < 0.05, *n* = 13), Thus, the above results suggest that relief of anxiety phenotype in VPA mice appears to be age-dependent.

### Overexpression of PKMζ in BLA Leads to Higher Anxiety and Elevated Intrinsic Excitability in the WT Mice

**Figure 4:**
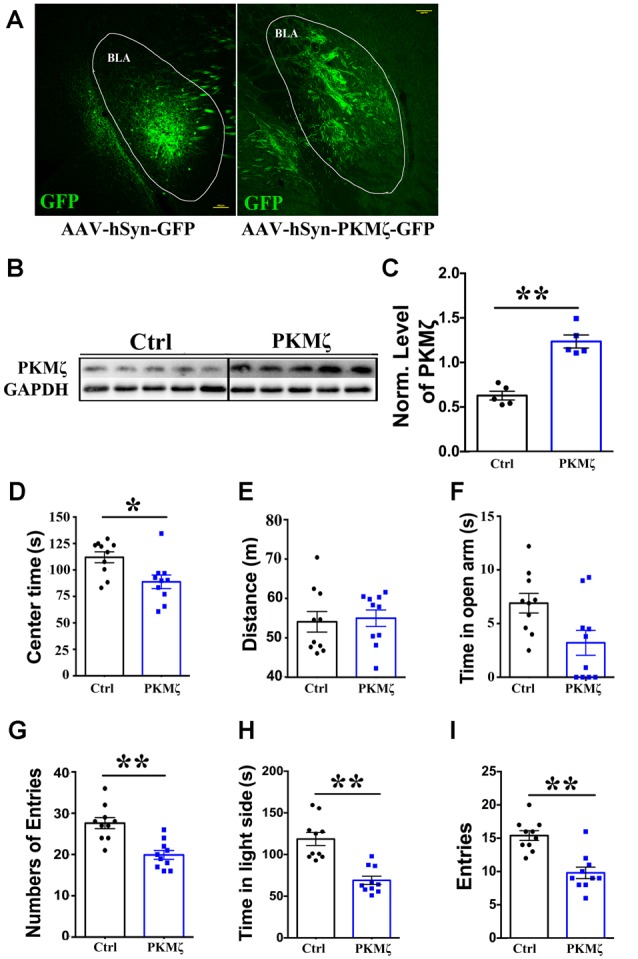
Overexpression of PKMζ led to higher anxiety in wild type (WT) mice. **(A)** Sample images showing overexpression of PKMζ or control virus in the BLA of P70 WT mice. Scale bars = 100 μm. **(B)** Sample images of WB analysis of PKMζ levels in the BLA from mice with either PKMζ overexpression or control virus. **(C)** Quantification showed significantly higher level of PKMζ in the BLA from mice injected with PKMζ virus compared to those injected with control virus. Time in the center **(D)** of open field test was significantly reduced in mice injected with PKMζ virus compared to those injected with control virus. Distance **(E)** traveled was not affected in mice injected with PKMζ virus compared to those injected with control virus. Time in the open arms **(F)** was not affected, numbers of entries to the open arms **(G)** was significantly decreased in the EPM in mice injected with PKMζ virus compared to those injected with control virus. Time in the light box **(H)** and entries to the light box **(I)** were both significantly decreased in mice injected with PKMζ virus compared to those injected with control virus. *P < 0.05, **P < 0.01.

Elevated PKMζ level in BLA and amelioration of anxiety in VPA mice by ZIP infusion suggested that BLA PKMζ plays a key role in the occurrence of anxiety in VPA model mice. If this is the case, we expect that increasing PKMζ in WT mice may result in elevated anxiety. To test this, we overexpressed PKMζ using AAV viral transfection in the BLA of P70 WT mice (Figure 4A; Scale bars = 100 μm). Western blot analysis confirmed the effectiveness of this manipulation by showing a significantly higher level of PKMζ in mice expressing AAV-hSyn-PKMζ-GFP compared to AAV-hSyn-GFP (Figures 4B,C; *P* < 0.01, *n* = 5 mice).

In mice with PKMζ overexpressed, we found significantly reduced center time in the OPT (Figure 4D, *P* < 0.05, *n* = 10 mice), distance traveled in OPT was not altered (Figure 4E; *P* = 0.25, *n* = 10), suggesting no alteration in locomotion. We found that time in the open arm not altered (Figure 4F; *P* = 0.87, *n* = 10) and significantly reduced numbers of entries to the open arms (Figure 4G; *P* < 0.01, *n* = 10) in the EPM. We also found that significantly reduced time in the light box (Figure 4H; *P* < 0.01, *n* = 10) and significantly reduced entries to the light box (Figure 4I; *P* < 0.01, *n* = 10) in the shuttle box test, in mice injected with PKMζ virus compared to those injected with control virus. Put together, these results prove strong evidence for elevated PKMζ level in BLA underlies the occurrence of anxiety.

**Figure 5:**
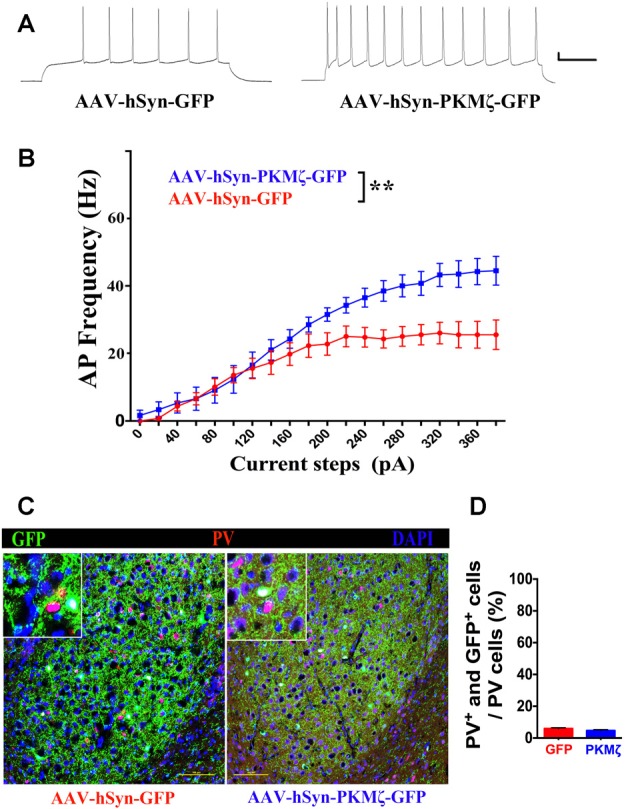
Overexpression of PKMζ resulted in higher intrinsic excitability in the excitatory neurons in BLA. **(A)** Sample spikes evoked by current injection showed higher responses in AAV-hSyn-PKMζ-GFP (right) than in AAV-hSyn-GFP (left) to the same current step (20 mV, 100 ms). **(B)** Current-spike frequency relationship showed higher responses in neurons expressing AAV-hSyn-PKMζ-GFP (blue) than expressing AAV-hSyn-GFP (red). **(C)** Sample staining of GFP and parvalbumin (PV) in sections from AAV-hSyn-GFP-expressing (left) and AAV-hSyn-PKMζ-GFP-expressing brains (right). Scale bars = 100 μm. **(D)** Density of neurons that are positive for both GFP and PV is very low. *P* = 0.1574, *N* = 5 sections/mice. **P < 0.01.

To further elucidate the cellular target of PKMζ, we measured intrinsic excitability in the excitatory neurons in BLA of P70 WT mice expressing AAV-hSyn-PKMζ-GFP or AAV-hSyn-GFP. By injecting a series of depolarizing current steps, we constructed the relationship between current steps and evoked spike frequency. As seen from sample traces (Figure 5A) and population data (Figure 5B), spike frequency is significantly higher in neurons from BLA with PKMζ overexpressed compared to neurons from mice injected with control virus (*P* < 0.01, *N* = 11 cells/5 mice). This result indicates that elevated PKMζ may increase the intrinsic excitability of BLA neurons which in turn drives higher anxiety. Since the average spiking frequency in neurons expressing AAV-hSyn-PKMζ-GFP reached about 40 Hz, we wanted to confirm that they were not the inhibitory PV neurons which are fast-spiking. We stained brain sections with PV (Figure 5C) and found very low overlapping between PV and GFP (Figure 5D), suggesting that they are not PV neurons.

## Discussion

In the present study, we used VPA ASD model mice to examine potential major molecular mechanism underlying the high anxiety phenotype in these mice, in an attempt to understand the genesis of anxiety in ASD. Our findings confirm high anxiety in VPA mice in both adolescence and adult, and indicate that this anxiety is associated with higher expression of PKMζ in the BLA.

Among the three major brain regions that contribute to anxiety, we found a selective increase in PKMζ level in the BLA, but not in HPC or mPFC. Interestingly, this increase is already significant at P35, the earliest time point, we have tested and still present at P70. This profile of expression suggests that PKMζ level in BLA might be an important contributor to certain phenotypes in the VPA mice. We have selected to focus on anxiety which is quite prominent at both P35 and P70, based on previous studies and our own findings. Supporting a critical role of PKMζ in anxiety in VPA mice, inhibiting PKMζ activity in BLA significantly reduces anxiety levels in P35 VPA mice. In addition, viral overexpression of PKMζ in BLA leads to elevated anxiety level in WT mice, which is associated with higher intrinsic excitability of BLA excitatory neurons. Thus, we have provided substantial evidence on the important contribution of PKMζ in BLA to the genesis of anxiety, in both normal mice and ASD model mice. Mounting clinical evidences indicate the prominent presence of anxiety in ASD patients (Ishimoto et al., 2019; Schiltz et al., 2019). We found that reducing PKMζ activity with ZIP peptide reduces anxiety in P35 VPA mice but not in P70 VPA mice. Since PKMζ level in BLA increases substantially during development and PKMζ level is relatively low at P35 compared to the P70 VPA mice, it is thus possible that locally administered ZIP can effectively inhibit PKMζ activity at P35 but not P70. In addition, anxiety in the P70 mice may involve more brain regions than the BLA, while BLA could have a more significant contribution to the genesis of anxiety at P35. It should be noted that there is some debate on whether at the concentration used, ZIP is selective for PKMζ (Lisman, 2012).

The highly elevated anxiety level in VPA mice allowed us to examine the cellular and molecular basis of this high anxiety. Hyperactivity in the BLA has been implicated in the genesis of anxiety. First, there is a negative correlation between GABAergic markers in the amygdala and anxiety level in that lower GABAergic markers are associated with higher anxiety, implicating an association between BLA hyperactivity and anxiety (Martijena et al., 2002; Flores-Gracia et al., 2010; Quadrato et al., 2014). Second, reducing BLA activity reduces anxiety. Barbalho et al. (2009) showed that infusion of benzodiazepine into BLA significantly reduced anxiety level in both EPM-naïve and EPM-experienced mice, while Sanders and Shekhar (1995) found that inhibiting GABAa receptors in the BLA elevated anxiety level. Third, Olexová et al. (2016) found an elevated mRNA level of type 1 GABA transporter in the BLA of VPA mice together with a high anxiety level. Whether GABAergic transmission is reduced in BLA in VPA mice needs to be tested directly. Banerjee et al. (2013) reported reducing inhibitory transmission in the cortex of VPA mice.

Reduced inhibitory function/transmission likely leads to hyperactivity in BLA, which has been reported in various psychiatric disease and stress models (Sharp, 2017). Another mechanism to cause hyperactivity is higher intrinsic neuronal excitability, as we have examined in this study. Since, we found elevated level of PKMζ in BLA, and there is evidence for PKMζ contributing to anxiety (reduced anxiety in the *Prkcz* null mice (Lee et al., 2013), although the brain regions involved and underlying mechanism not examined), we tested whether there is a link between PKMζ level and hyperactivity in the BLA neurons. We showed that overexpression of PKMζ leads to higher excitability in BLA excitatory neurons in WT mice, and hence potentially linking PKMζ level in BLA neurons to anxiety. Overexpression of PKMζ in the P70 WT mice led to elevated anxiety in them (Figures 4D–I). Thus, these collected evidences provide substantial support for the selective contribution of BLA PKMζ to anxiety. Another way that PKMζ may lead to hyperactivity is that it may result in stronger synaptic connections to BLA neurons, as Shuette (Chihabi et al., 2016) showed that overexpressing PKMζ increased basal synaptic transmission mediated by increased AMPA receptor trafficking.

What leads to elevated PKMζ level in VPA mice? Many studies have shown the importance of PKMζ in the formation and especially maintenance of memory, such as spatial memory and fear memory (Volk et al., 2013). For example, Holliday et al. (2016) showed that reconsolidation of fear memory is associated with elevated level of PKMζ in the BLA. PKMζ in BLA is also required for the maintenance of contextual fear memory (Kwapis et al., 2009; Volk et al., 2013). Interestingly, Xue et al. (2015) reported that overexpression of PKMζ in the prelimbic PFC facilitates the formation of fear memory. Hence, there is a possibility that the more prominent presence of aversive memory (such as fear memory) in VPA mice is caused by elevated PKMζ level in BLA of VPA mice, and accumulation of these adverse events may result in high anxiety. In a single prolonged stress model of post-traumatic stress disorder, Ji et al. (2014) showed that infusion of ZIP effectively reduced anxiety and depression in these mice, consistent with the notion that PKMζ contributes to elevated anxiety caused by stress. Thus, there is a possibility that heightened sensitivity to stress may lead to a higher level of PKMζ in VPA mice and anxiety, but this hypothesis needs to be tested directly.

## Data Availability Statement

The datasets generated for this study are available on request to the corresponding author.

## Ethics Statement

All animal experiments and procedures were approved by the Ethics Committee of Peking University (Permit Number: AP0011).

## Author Contributions

QZ, XG and RZ conceived and designed the experiments. RZ analyzed the data and drafted the manuscript. XM, XG, ZG and DX performed the experiments and helped to revise the manuscript. All authors read and approved the final manuscript.

## Conflict of Interest

The authors declare that the research was conducted in the absence of any commercial or financial relationships that could be construed as a potential conflict of interest.
